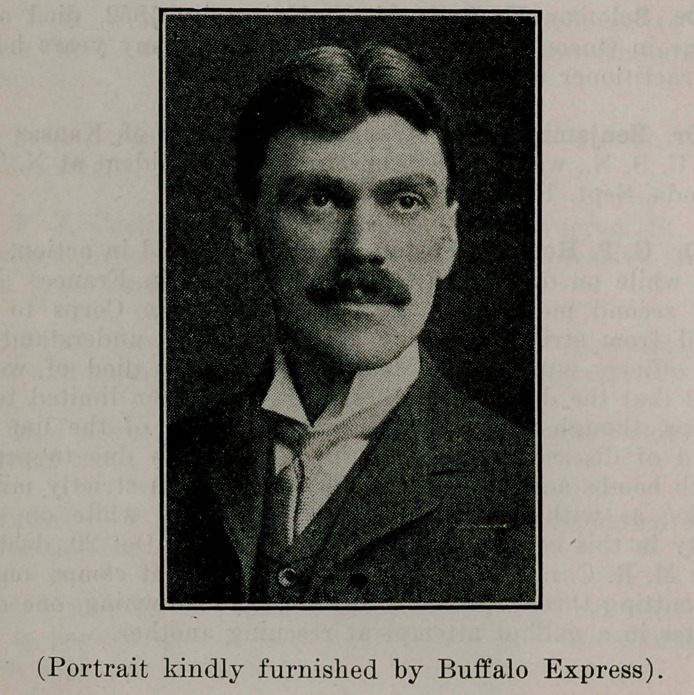# Dr. James A. Gibson

**Published:** 1917-11

**Authors:** 


**Dr. James A. Gibson,** Western University of London, 1890, died at his home in Buffalo Oct. 4, aged 50, of pneumonia, complicated with cardiac disease. He was born at London, Ont., June 3, 1867, and received his collegiate education at the University of Toronto. He came to Buffalo 25 years ago and always took a prominent part in professional activities. He was a member of the old Fitch Hospital staff, since 1893, of the Roswell Park Medical Club, has been Prof. of Anatomy and Secretary of the Faculty in the Medical Dept., University of Buffalo, since 1910 and was affiliated with the various local and general medical societies. He was an honored member of the Nu Sigma Nu Fraternity and was a Mason in high standing. The funeral was held at Calvary Church, Sunday, Oct. 7, being attended by large delegations from the Medical Society of the County of Erie and the Buffalo Academy of Medicine, the students and the Masonic body, the Roswell Park Medical Club, the Faculty and other organizations. Dr. Gibson had the force and efficiency often attending quiet and modest natures and was beloved by all.

## Figures and Tables

**Figure f1:**